# Research review of the mechanism and clinical application prospects of tertiary lymphoid structures in the immune micro-environment of gastrointestinal tumors

**DOI:** 10.32604/or.2025.058957

**Published:** 2025-06-26

**Authors:** JIANG ZHU

**Affiliations:** Second Clinical Medical School, Nanjing Medical University, Nanjing, 210000, China

**Keywords:** Tertiary Lymphoid Structures (TLS), Gastrointestinal tumors

## Abstract

Changes in the intestinal immune micro-environment of the gastrointestinal tract are indispensable in the occurrence and development of gastrointestinal cancer. Tertiary lymphoid structure (TLS) is an immune cell aggregation structure found around gastrointestinal cancer in recent years. More and more research proves that tertiary lymphoid structure plays a key biological role and clinical value in disease progression, patient prognosis, and adjuvant treatment. This review aims to explore the research progress, biological significance, and potential clinical applications of TLSs in gastrointestinal tumors. The formation, development, and interaction of TLSs with tumor microenvironment have been reviewed and analyzed in recent years. Meanwhile, this review not only evaluates the clinical value of TLSs as prognostic biomarkers and predictors of treatment response but also explores their role in guiding the formulation of immunotherapy strategies for gastrointestinal tumors. In addition, this review points out the main problems in the current research of TLSs and looks forward to their future development, especially their broad application prospects in the diagnosis, treatment, and prognostic evaluation of gastrointestinal tumors.

## Introduction

Gastrointestinal tumors refer to tumors that occur in the gastrointestinal tract such as the stomach, small intestine, and large intestine. As one of the tumors with high incidence, they pose a serious threat to the health of people all over the world. According to the World Health Organization, the incidence and mortality of gastric cancer in 2022 ranked fifth and fourth among malignant tumors in the world, respectively. As one of the countries with a high incidence of gastric cancer in China, its new cases and deaths account for about half of the total worldwide. The incidence of colorectal cancer ranks third among malignant tumors in the world, and the mortality rate ranks second [1]. In terms of treatment, gastrointestinal tumors face many challenges. The early symptoms of gastrointestinal tumors are not apparent, and many patients are already in the middle and late stages when they are diagnosed. Many studies have shown that B cells and tertiary lymphoid structures (TLS) are related to better prognosis of patients with different tumors [2], but the specific mechanism of TLS shaping the tumor micro-environment and exerting anti-tumor effects is still unclear. Hence, an in-depth analysis of TLS helps to fully tap its potential application value in anti-tumor immune response and guide precise immunotherapy of tumors.

TLSs, also known as tertiary lymphoid tissue or ectopic lymphoid tissue, are organized immune cell aggregates formed in non-lymphoid organs under a pathophysiological environment, especially under the stimulation of chronic inflammation (such as tumors). Mainly composed of B cells, T cells, dendritic cells, stromal cells, and other immune cells, these structures contain structures such as high endothelial venules (HEV), which contribute to the migration and localization of lymphocytes [3]. Specifically, TLSs serve as a key platform for the confrontation between immune cells and tumor cells in the tumor immune microenvironment (TIME), and their existence is directly related to the patient’s prognosis assessment and the intensity of response to immunotherapy. For example, the infiltration of PD-1 + CD8+ T cells located in TLS can predict the blocking response of PD-1 in patients with advanced non-small cell lung cancer [4]. According to previous studies, the effect of TLS on immunotherapy such as PD-(L) 1 or PD-(L) 1 combined with cytotoxic T lymphocyte-associated protein 4 (CTLA-4) blockade is mostly positive, which has been demonstrated in multiple immunotherapy cohorts such as melanoma [5] and bladder cancer [6].

## Overview of Tertiary Lymphoid Structures

**Figure 1:**
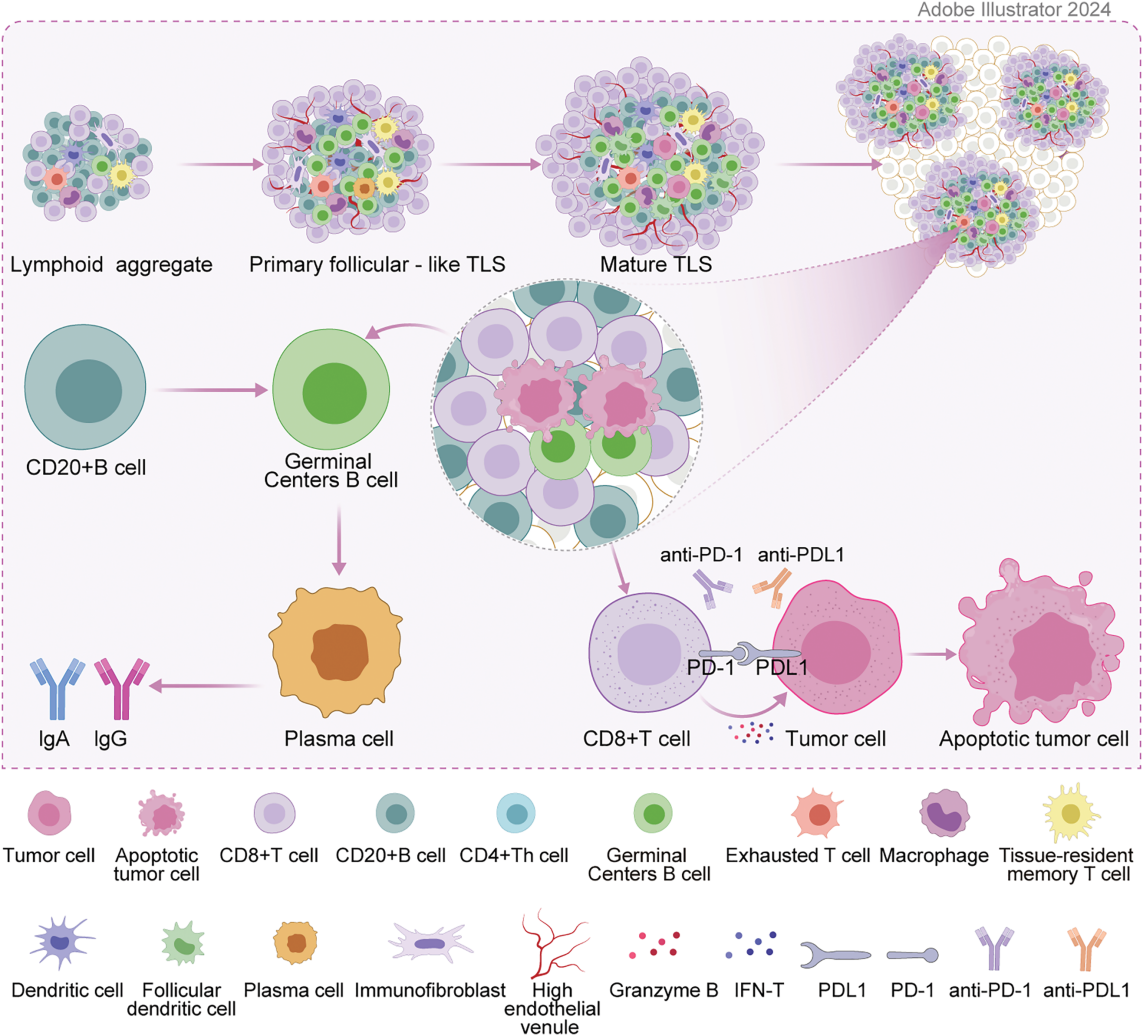
Formation of TLS and mechanisms of tumor immunity.

TLSs are significantly different from congenital primary lymphoid organs (thymus, bone marrow) and secondary lymphoid organs (spleen, lymph nodes). They are mainly formed under the stimulation of chronic inflammation (especially tumor and infection environment), and their structure and function highly mimic secondary lymphoid structures. The formation of TLSs is a complex and delicate process of cell-molecule interaction. Similar to Secondary Lymphoid Organs (SLO) formation, under inflammation, the inflammatory factors interleukin-13 (IL-13) or IL-17 drive tissue fibroblast activation, and IL-22 promotes tissue fibroblast expansion. They jointly recruit and activate tissue fibroblast induction into lymphoid tissue inducers (LTi) under the action of CXC-chemokine ligand 13 (CXCL13) and IL-7 [7]. LTi cells interact with stromal cells through the binding of lymphotoxin-α1β2 ligand (LTα1β2) to lymphotoxin beta receptor (LTβR). In addition, they jointly induce the secretion of chemokines, angiogenic growth factors, and the expression of cell adhesion molecules [8]. The expression of some of these factors is further amplified by interaction with other cell types (e.g., dendritic cells, CD8 T cells, or natural killer cells), ultimately triggering the secretion of Vascular Endothelial Growth Factor A(VEGFA), Vascular Endothelial Growth Factor C (VEGFC) and promoting High endothelial venules (HEV) formation. CXCL12 and CXCL13 are also produced. Chemokines CC-chemokine ligand 19 (CCL19) and CCL21 are for immune cell recruitment, expressing vascular cell adhesion molecule 1 (VCAM1) and intercellular adhesion molecule 1 (ICAM1) to guide immune cell colonization to corresponding sites [9]. Meanwhile, the cytokine IL-36γ secreted by macrophages and endothelial cells can promote the ability of HEV to recruit lymphocytes by up-regulating the expression of VCAM1 and ICAM1 in stromal cells and vascular endothelial cells, and further enhance the formation and maturation of TLS [10]. The last stage of TLS maturation is the formation of follicular dendritic cells (FDCs) in B-cell follicles. The precursor cells of FDCs in B cell follicles form FDCs under the action of the lymphotoxin-β receptor (LTβR) signaling [11], thus a mature TLS is formed [12]. Fig. 1 depicts the formation of TLS and the mechanisms of tumor immunity. In terms of cellular composition, it is similar to SLO, including adaptive lymphocytes, plasma cells, germinal center, dendritic cells (DC), follicular dendritic cells, stromal cells, high endothelia [13] and lymphatic vessels [13,14]. According to the cell aggregation region, TLS can be further divided into the T cell region, B cell region, and HEV. The T cell region is characterized by CD3+ T cells and mature DC (DC-Lamp+) clusters. The B cell region consists of an active germinal center [15]. HEV expresses peripheral node addressing protein (PNAd) with the function of recruiting lymphocytes in the blood. In the gastrointestinal tumor environment, TLS formation is influenced by a variety of factors, including immune cells and their interactions, cytokines and chemokines, the tumor microenvironment, and other factors such as gut microbes and genetic epigenetic factors. These factors interact to jointly regulate the formation and development of TLSs, thereby affecting the efficacy of anti-tumor immune response and patient prognosis.

## Research Progress of TLSs in Gastrointestinal Tumors

### Key biological role of TLSs in the occurrence and development of gastrointestinal tumors

TLSs play an important role in the immune response of gastrointestinal tumors. As ectopic lymphoid structures in non-lymphoid tissues, they promote the aggregation and activation of immune cells. TLSs are involved in the anti-tumor response through both cellular and humoral immunity. In terms of cellular immunity, T cells within TLSs (such as CD8+ cytotoxic T cells) can recognize and kill tumor cells. In terms of humoral immunity, B cells develop within TLSs and produce antibodies that help clear tumor cells or inhibit their growth through humoral circulation. The presence of TLSs is strongly associated with better prognosis and immunotherapy response in patients with gastrointestinal tumors. In TLSs, different cell subsets act synergistically to enhance the immune response [16]. Follicular dendritic cells are located in the center of B cells, express various receptors, promote antigen presentation to B cells, drive B cell activation, and produce highly active antibodies. T cell subsets such as CD4+ helper T cells (Th) and CD8+ cytotoxic T cells are involved in immune regulation and direct killing of tumor cells, respectively. Meanwhile, CD4+ Th cells promote B cell antibody production and CD8+ T cell activation by secreting cytokines, while CD8+ T cells recognize tumor antigens and elicit cytotoxic responses. The synergistic action of these cell subsets constitutes the complex immune network in TLSs, which is crucial for anti-tumor immune responses. Other components within TLSs have also been found to have positive prognostic value, including HEVs [17], chemokine-12 [18], CD3+ T cells [19], and CXCL13 [20]. Specifically, CXCL13 of B cells, as a product of Follicular helper T cell(Tfh), is intertwined with the formation of TLSs and suggests a good prognosis [21]. In addition to CXCL13 involved in inducing the formation of TLSs, CXCL12 was also found to be closely related to the formation of TLSs, and the expression of CXCL12 features could effectively promote the enrichment of TLSs in the tumor microenvironment. Moreover, the enrichment of TLSs is positively correlated with the number of mutations/neoantigen burden. According to the mutation information obtained from the DNA sequencing data of The Cancer Genome Atlas (TCGA) database, melanoma, lung squamous cell carcinoma, and lung adenocarcinoma tumors with a high number of mutations or neoantigen burden show highly expressed TLS characteristics. Studies have shown that the clinical response of patients with advanced cervical intraepithelial neoplasia (the early stage of cervical cancer) to the human papillomavirus (HPV) vaccine is related to the induction of TLSs into tumors [22]. Thus, some research teams have proposed the idea of locally inducing the formation of TLSs by injecting related chemokines (CXCL13, CCL19, and CCL21) at corresponding positions [23]. However, there is still a lack of research to verify this assumption.

The density, location, maturity, and other characteristics of TLSs are intertwined with the prognosis of patients with gastrointestinal tumors. Several studies have evaluated the value of TLS in predicting the prognosis of patients with colorectal cancer, given that TLS is associated with a low local recurrence rate, low distant metastasis rate, and high survival rate [24]. In colorectal cancer, TLS density has been reported to be positively associated with better survival and negatively associated with tumor stage [25,26]. The high density of proximal breast TLS is related to the decreasing disease-free survival (DFS) but not overall survival (OS), while the density of distal breast TLS is negatively correlated with both DFS and OS [27]. A Western study on TLS and gastric cancer [28] found that the survival rate was 28.6% in the low TLS density group and 35.6% in the high TLS density group. Compared with the low TLS density, patients with high TLS density had a longer 5-year survival rate. The 5-year survival rate of the low maximum TLS diameter group was 26.4%, and that of the high maximum TLS diameter group was 37.8%. Compared with the low maximum TLS diameter, the high maximum TLS diameter was associated with a longer 5-year survival rate. Whereas in the subgroup analysis of intestinal type histology, in the diffuse subgroup, high maximum TLS diameter was associated with improved 5-year survival and improved overall survival compared with low maximum TLS diameter. According to a recent large study in China that included 914 patients with gastric cancer, high-density TLS was associated with a good prognosis. In a few small studies based on immunohistochemistry, a large number of TLS were also associated with a good prognosis of gastric cancer [29,30]. TLS density and diameter are related to the prognosis of gastric cancer, which may be related to the anti-tumor immune response reflected by TLS. Within TLS, dendritic cells introduce antigens into CD4T cells, resulting in T-cell activation and B-cell maturation. At the same time, TLS size and density may be related to the maturity of TLS, which may result in a good prognosis. It has been demonstrated in lung squamous cell carcinoma and colorectal cancer [31,32]. Besides, TLSs with high maturity, such as mature follicles containing germinal centers, are associated with better prognosis because they can more effectively promote the activation and differentiation of immune cells. Primary and secondary follicles are defined as immature TLS, while lymphoid aggregates are defined as mature TLS [33,34]. One study [35] included 292 patients with gastric cancer in immature TLS. Compared with patients with low TLS, the prognosis of patients with high TLS was not significantly improved. In mature TLS, compared with patients with low TLS, the prognosis of patients with high TLS is significantly improved. Furthermore, mature TLS was significantly increased in the mixed and intestinal types compared to diffuse gastric cancer in the Lauren classification. Compared with the middle and distal cases, the mature TLS in the proximal cases also increased significantly. It was found that the cellular components in mature TLS and immature TLS showed obvious differences. The number of CD8 T cells and CD20 B cells increased greatly in mature TLS, but no differences were observed in immature TLS. The study also found that positive immune cells of Programmed Cell Death Protein 1 (PD1) are mostly located around mature TLS, the number of PD1CD8 T cells and the expression of Programmed Cell Death Ligand 1 (PD-L1) on tumor cells is also significantly increased. As prognostic biomarkers, TLSs have shown significant evidence in multiple studies. From January 2014 to July 2017, a retrospective analysis of 203 tissues with pathological diagnosis of Colorectal cancer after general surgery was conducted in the First Affiliated Hospital of Jinan University. It was found that patients with high endothelial vein (HEV)/TLS in Colorectal cancer (CRC) tissues were associated with DFS and lower TNM stages [36]. At the same time, CRC tissues with high HEV/TLS have stronger recruitment capacity for CD3+ T cells, CD8+ T cells, and M1 macrophages, which are associated with less angiogenesis. In conclusion, high HEV/TLS is associated with good prognosis in CRC patients and anti-tumor immune microenvironment, which can be used as a potential biomarker of prognosis in CRC patients. The density, location, and maturity of TLSs are positively correlated with the prognosis of patients with various gastrointestinal tumors. High density, high maturity, and the presence of TLSs around tumors often predict better clinical outcomes. These findings provide strong support for TLSs as potential prognostic markers [32].

In recent years, remarkable progress has been made in the mechanism of TLSs in the occurrence and development of gastrointestinal tumors. As aggregates of immune cells formed in non-lymphoid tissues, TLSs play a vital role in gastrointestinal tumors. Its formation usually begins with the immune response caused by chronic inflammation or tumor, which promotes the aggregation and interaction of immune cells. Studies have indicated that the density and maturity of TLSs are intertwined with the prognosis and immunotherapy response of gastrointestinal tumors. The high density of TLSs predicts a better prognosis because of their ability to activate anti-tumor immune responses more efficiently [26]. Moreover, immune cells within TLSs, such as B cells and T cells, further promote the recruitment and activation of immune cells by secreting cytokines and chemokines, thereby enhancing the anti-tumor effect. Evidence shows that TLSs are positively associated with lymphocyte infiltration in gastric cancer [37], and the formation of TLSs predicts favorable immune system function. However, the specific mechanism of action of TLSs in gastrointestinal tumors has not been fully elucidated. Although there is a certain association between TLSs and gene mutations and epigenetic changes in gastrointestinal tumors, the specific mechanism has not been completely clear. Studies have shown that the formation of TLSs may be regulated by gene mutations and epigenetic changes. Some gene mutations may promote the expression of chemokines and cytokines, thus inducing the formation of TLSs. For example, Wang et al. screened out seven TLS-related genes ADAM8, SLC6A1, PAXX, RIMKLB, PTH1R, CD1B, and MMP10 to construct a prognostic model [38]. Survival analysis proved that the overall survival rate of patients in the high-risk group was significantly lower. The immune micro-environment analysis indicated that the high-risk group patients had higher immune indexes with higher immunity. There are significant differences in genomic mutation patterns between high-risk and low-risk groups, especially since frequency of KRAS mutation in the high-risk group is significantly higher than that in the low-risk group.

### Clinical value of TLSs in gastrointestinal tumors

PD-L1 IHC 22C3 pharmDx is an immunohistochemistry test kit used to detect the level of PD-L1 expression in tumor tissue. Generally, the testing fee ranges from several thousand to several ten thousand yuan. CPS (Combined Positive Score) is a scoring system for evaluating PD-L1 expression level, which combines the expression of PD-L1 on tumor cells and immune cells to provide doctors with key information about whether a patient is suitable for receiving specific immunotherapy (such as anti-PD-1/PD-L1 therapy). Anti-PD-1/PD-L1 therapy is an immunotherapy that blocks the interaction between PD-1 and PD-L1 to restore the killing function of T cells against tumor cells and thus inhibit the growth and spread of tumors. There are currently several anti-PD-1/PD-L1 drugs on the market, such as pembrolizumab (Keytruda, K drug), nivolumab (Opdivo, O drug), and tremelimumab.

Table 1 summarizes the clinical value of various cells in TLS for a more intuitive understanding of the clinical value of TLS for tumors. TLSs in tumor microenvironment have been proven to be a vital component of immunotherapy and are intertwined with the efficacy of immunotherapy [39]. ICIs of anti-CTLA-4 and anti-PD-1 antibodies work by promoting T cell activation and are currently mainly used to treat metastatic melanoma. It was found that high levels of TLSs correspond to longer OS in melanoma patients receiving anti-CTLA-4 immunotherapy [40]. Studies on melanoma patients receiving anti-PD-1 immunotherapy also reached the same conclusion [41,42]. It also suggests that TLSs are closely related to the prognosis of immunotherapy in melanoma patients. In addition, it is also found in lung cancer that the response of TLSs with high scores to immunotherapy will be improved. There is evidence that there is a positive humoral anti-tumor response driven by B cells in TLSs. However, the exact role of B cells, and TLSs in particular, remains unclear on the response to ICIs [43,44]. Also critical to the immunotherapy of gastrointestinal tumors, TLSs serve as a platform for immune cell aggregation and can enhance immune responses. In PD-1/PD-L1 inhibitor therapy, the presence of TLSs may predict a better therapeutic response because they promote T cell activation and anti-tumor immune response. CD103+ T cells are located around TLS. Patients with high expression of CD103 have more abundant TLS, better prognosis, and better response to PD-1 blocking therapy [45]. TLS scores are associated with immunotherapy-related characteristics, such as microsatellite instability and tumor mutation load. Patients with higher TLS scores are characterized by prolonged survival and superior response to immunotherapy. Furthermore, RNA-seq data analysis of the Zhongshan immunotherapy team showed that higher TLS scores were associated with favorable responses to PD1 blocking therapy [35]. For cell therapies such as CAR-T therapy, TLSs may provide a favorable environment to promote the infiltration and proliferation of CAR-T cells, thus enhancing the therapeutic effect. At present, there is no clear conclusion about the mechanism of TLS promoting the efficacy of immunotherapy in esophageal squamous cell carcinoma. There is a special subset of CD8+ T cells in TLS in the tumor microenvironment, called progenitor-exhausted T cells (Tpex). Under the long-term stimulation of tumor antigens, some tumor-infiltrating lymphocytes will up-regulate the expression of some immunosuppressive receptors, such as PD-1, TIM3, LAG-3, and TIGIT. This part of cells is called exhausted T cells (Tex) [46]. Tex subsets are the predominant cell types that respond to PD-1/PDL1 immune checkpoint inhibitors, and immunotherapy is able to reactivate Tex to exert its anti-tumor effects [47,48]. However, the Tex subset itself is heterogeneous, including two subsets of Tpex and terminal exhausted T cells (Ttex). The Tpex subset is the key cell population to maintain the long-term immune response mediated by CD8+ T cells and respond to immunotherapy [49–53]. PD-1/PD-L1 pathway blockade mainly acts on Tpex subsets, allowing them to further differentiate into downstream cell subsets, namely Ttex subsets [47,48,50,54]. The higher the proportion of Tpex subsets in the tumor microenvironment, the better the prognosis of tumor patients after immunotherapy [55–57]. TLS in the tumor microenvironment may help Tpex to accumulate in the tumor, thereby enhancing the efficacy of immunotherapy [4,58,59]. In esophageal squamous cell carcinoma, recent studies [60] have found that a population of Tpex-like CD8+ T cells can enhance the tumor’s response to immunotherapy. This population of cells also can be used as an effective predictor of the efficacy of immunotherapy. In addition, the researchers found that this group of cells is also co-localized with B cells in the TLS structure, which has a strong cell interaction relationship with TLS-related B cells. This study suggests that Tpex subset cells may be an important target cell population for TLS to promote esophageal squamous cell carcinoma in response to immunotherapy. The characteristics of TLSs significantly affect the efficacy of immunotherapy. The density, location, and maturity of TLSs are key factors. The high density of TLSs indicates a stronger immune response, which may improve the effect of immunotherapy. TLSs are located around or inside tumors, directly affecting the interaction between immune cells and tumor cells. Highly mature TLSs contain germinal centers, which can more effectively promote the activation and differentiation of immune cells. Based on these clinical values of TLS, we can directly inject specific cytokines (such as CXCL13 and CCL21) into the tumor to promote immune cell infiltration, TLS formation, and tumor shrinkage. We can also combine cell factors with tumor vaccines and other treatments to stimulate TLS formation, reduce the generation of immunosuppressive cells (such as Regulatory T cells), and improve patient survival rates. For example, nano-vaccine technology can use specific delivery vectors and adjuvants to deliver antigens to the tumor site while stimulating TLS formation, further enhancing anti-tumor immune responses [16]. We can also modify cells to enable them to produce large amounts of relevant cytokines, and then inject these cells into the tumor to induce TLS formation, increase immune cell infiltration, and improve anti-tumor responses.

**Table 1:** Clinical value of TLS

TLS	Effect
T cell	Immune checkpoint inhibitors (ICIs) of anti-CTLA-4 and anti-PD-1 antibodies work by promoting T cell activation
B cell	Positive humoral anti-tumor response driven by B cells
Tpex	Maintain CD8+ T cell-mediated long-term immune response and a critical cell population in response to immunotherapy
Density	A high density of TLSs indicates a stronger immune response and may enhance immunotherapy efficacy
Location	TLSs are located around or inside the tumor and directly affect how immune cells interact with tumor cells
Maturity	TLSs with high maturity contain germinal centers, which can promote the activation and differentiation of immune cells more effectively
CD3+, CD20+	As an immune cell marker in TLSs, the composition and maturity of TLSs can be evaluated through immunohistochemical detection, so as to judge the immune microenvironment of tumors

The expression of TLSs in patients with gastrointestinal tumors is complex and diverse. Studies have shown that TLSs occur in a variety of gastrointestinal tumors. There may be differences in the composition, density, and distribution characteristics of TLSs in various pathological types of gastrointestinal tumors. For example, in gastric cancer, the degree of invasion of TLSs may be closely related to the patient’s prognosis. The higher the density and degree of invasion of TLSs, the better the patient’s prognosis [30,61]. However, the current research on the specific expression characteristics of TLSs in different pathological types and stages of gastrointestinal tumors is insufficient. More clinical and experimental data are needed to support it. As a predictor of prognosis or treatment response, TLSs have manifested important clinical value in tumors. For example, in high-grade serous ovarian cancer, the appearance of mature TLSs (mTLS) is associated with a better prognosis and increased sensitivity to immune checkpoint inhibitor therapy. The density of TLS is key to controlling the occurrence and development of tumors, which can be used as one of the parameters of colorectal tumor progression. It is defined as the ratio of the number of follicles to the length of the leading edge of invasion. TLS density was positively correlated with better survival and negatively correlated with tumor stage. Meanwhile, as another crucial parameter to evaluate the prognostic value of patients, TLS maturity is mainly based on the proportion of early TLS (E-TLS), primary follicular TLS (PFL-TLS), and secondary follicular TLS (SFL-TLS). Its predictive ability for colorectal cancer recurrence may be higher than TLS density [32]. Studies have shown that the risk of recurrence in patients with SFL-TLS is significantly lower than in patients without any GC-TLS (9.5% *vs*. 28.5%) [32]. This indicates that TLS with GC response represents a “functional” subtype, and the development of GC indicates the initiation of tumor-specific B cells and CD4+ T cells. Furthermore, the immune cell composition of TLSs, such as the distribution of B cells and T cells, can be used as a predictor of immunotherapy response. These findings suggest the importance of TLSs in assessing patient prognosis and guiding individualized treatment decisions.

TLSs have potential applications in the diagnosis of gastrointestinal tumors as immune hotspots in tumor microenvironment. Their existence, density, and maturity may reflect the immune status and prognosis of tumors. During the diagnosis, pathological diagnosis methods based on TLSs may become a new auxiliary means. By detecting immune cell markers in TLSs, such as CD3+, CD20+, etc., immunohistochemistry and other techniques, the composition and maturity of TLSs can be evaluated, so as to judge the immune micro-environment of tumors [62]. Such a method is helpful in understanding the biological characteristics of tumors more comprehensively, which provides a basis for formulating individualized treatment plans. TLS is an important marker reflecting the tumor microenvironment. Studies have shown that TLS can enhance the efficacy of immunotherapy in a variety of tumors, including melanoma [5,63], soft tissue sarcoma [64], lung cancer [65], etc. More studies have begun to explore the possibility of using TLS as a marker of immunotherapy efficacy. In the PEMBROSARC study [66], researchers explored the effect of using TLS as a marker to screen patients with soft tissue sarcoma to receive immunotherapy. The study included 35 patients with soft tissue sarcoma who were confirmed to have TLS before receiving immunotherapy and 41 patients who were not screened for TLS during the same period. The results of the study found that compared with patients who were not screened for TLS, the objective response rate (ORR) and PFS of TLS-positive patients were significantly increased or prolonged (30% *vs*. 2%, 4.9 months *vs*. 1.5 months, respectively). In esophageal squamous cell carcinoma, no related research has been carried out using TLS as a biomarker, but it has been reported that its immunotherapeutic efficacy is related to TLS. A study of 34 patients with recurrent esophageal cancer after surgery [67] found that patients with higher TLS density in the primary tumor had significantly prolonged PFS after immunotherapy alone compared with patients with lower TLS density [HR = 3.19, 95% CI (1.36, 7.43), *p* = 0.004]. TLS (Tertiary Lymphoid Structures) has good biomarker characteristics and risk reduction advantages in gastric and intestinal malignant tumors. TLS is associated with good prognosis and improved response to immunotherapy. The TLS scoring system may serve as a supplement to the current cancer staging system, providing doctors with more refined risk stratification information and helping to develop more personalized treatment plans. At the same time, the presence and characteristics of TLS may guide the selection and efficacy assessment of immunotherapy. For example, patients with rich TLS in ICI treatment may be more likely to achieve treatment effects, while patients with a lack of TLS or unclear features may need other treatment options [37].

In the aforementioned studies, inconsistent findings of TLS as a prognostic factor exist, which may be due to various factors, including differences in study design, sample size, and tumor type. Different studies may use different evaluation criteria and indicators to measure the prognostic value of TLS. For example, some studies may focus on the density, maturity, or composition of specific immune cell subpopulations, while others may focus on the overall structure and function of TLS. These different evaluation criteria may lead to different interpretations of the prognostic value of TLS. The size of the sample is an important factor affecting the consistency of research results. A smaller sample size may lead to unstable results because a smaller sample may not be able to adequately represent the entire patient population. This may result in differences in the prognostic value of TLS observed in different studies. The heterogeneity of tumor types is an important factor affecting the evaluation of TLS prognostic value. Different tumor types may have different immune microenvironments and TLS features. For example, some tumors may be rich in B cells and T cells, while others may be rich in other types of immune cells. These different immune microenvironments and TLS features may lead to different interpretations of the prognostic value of TLS.

However, at present, the specific application of TLSs in the diagnosis of gastrointestinal tumors is still in the research stage and needs further clinical validation and optimization. As immune hotspots in tumor microenvironment, the density, location, and maturity of TLSs can affect the efficacy of immunotherapy. High-density and mature TLSs can predict a better therapeutic response because of their efficient activation of anti-tumor immune responses. Therefore, when formulating immunotherapy strategies, the characteristics of patients’ TLSs should be evaluated. For patients with abundant and mature TLSs, the use of immunotherapies such as immune checkpoint inhibitors can be preferred. In addition, by promoting the formation and maturation of TLSs, such as the use of biological agents like cytokines, it is possible to enhance the immunotherapeutic effect and achieve personalized treatment.

## Existing Problems and Challenges

The current insufficient samples of TLSs in the study of gastrointestinal tumors and the limited number of samples used to define and characterize TLSs in different studies have limited the in-depth understanding and widespread application of their mechanism of action in gastrointestinal tumors. Although the presence of TLS is largely associated with prolonged patient survival, studies have found that TLS may be a negative prognostic factor [68]. These inconsistencies in the findings of different tumors may be related to the location of TLS, the heterogeneity of diagnostic methods, and the diversity of their immune cell composition. In the study of Lin et al. [69], DNA repair, tumor suppressor genes, genes involved in exogenous apoptosis, antigen presentation, and immunomodulation were found to be closely related to TLS in tumors. These findings all indicate the interaction between TLS in TME and carcinogenic changes in tumor cells. The detailed mechanism of their interaction also remains to be elucidated by further research. The underlying mechanisms of the development of TLS in some patients remain unknown, which may be due to the accumulation of aspects such as genetic, epigenetic, and transcriptional alterations as well as differences in molecular characteristics of tumors [70]. The formation mechanism and function of TLSs in gastrointestinal tumors and their relationship with tumor-specific immune responses are still incompletely understood, with further experimental studies and clinical validation needed. By integrating knowledge and technologies from multiple fields such as immunology, oncology, and pathology, and collaborating across disciplines, the complex mechanism of action of TLSs in gastrointestinal tumors can be more comprehensively revealed. The application of new technologies can also promote the in-depth research of TLSs. For example, single-cell sequencing technology can analyze the gene expression profiles of different immune cells in TLSs and provide data support for precision treatment [71]. High-resolution imaging technology is helpful in observing the dynamic changes of TLSs and revealing their interaction with tumor microenvironments. In addition, with the development of artificial intelligence and big data, the establishment of biomarker discovery and prediction models based on TLSs will also become possible, providing new ideas and methods for the prevention, diagnosis, and treatment of gastrointestinal tumors.

## Conclusion and Prospect

The research progress of TLSs in gastrointestinal tumors has revealed their importance in tumor immunity. As a key component in the tumor microenvironment, TLSs not only promote the anti-tumor immune response but also exhibit great potential as prognostic biomarkers and predictors of treatment response. In the future, with the strengthening of interdisciplinary cooperation and the application of new technologies, the application prospect of TLSs in the diagnosis, treatment, and prognosis evaluation of gastrointestinal tumors will be broader.

## Data Availability

Data sharing not applicable to this article as no datasets were generated or analyzed during the current study.
